# New Appliances and Things Medical

**Published:** 1910-02-12

**Authors:** 


### NEW APPLIANCES AND THINGS MEDICAL.

**[We shall be glad to receive at our Office, 28 & 29 Southampton Street, Strand, London, W.C., from the manufacturers, specimens of all new preparations and appliances.]**

### GUAIACOSE.

(The Bayer Co., Ltd., 19 St. Dunstan's Hill, London, E.C.)

This compound, a sample of which has been submitted to us for report, is described as a 5 per cent. solution of guaiacol calcium sulphonate in liquid somatose. The peculiar merit of this compound of guaiacol is that it is soluble, non-irritant, and non-toxic; nor does it convey the somewhat repulsive taste of the ordinary preparations of guaiacum. The calcium salt has been preferred to that of potassium on account of its less depressing action. Somatose, the vehicle for the drug part of the combination, is too well known to require description now; the large albuminous content of this solvent renders it a valuable food, so that the tonic and curative properties of the guaiacol are materially helped by the fluid in which it is administered. The dose of the mixture is one teaspoonful, and it is highly recommended for all pulmonary and bronchial conditions, from a common cold up to pulmonary tuberculosis. It would be of interest to ascertain what influence it manifests over those forms of osteoarthritis in which guaiacol carbonate so often does good; for these, as is well known, occur frequently in patients whose nutrition is very distinctly below par. The preparation is not unpalatable; it is made in Germany, and it costs 2s. 9d. per bottle of about four ounces.

### RILEY'S BILLIARD TABLES.

(E. J. Riley, Ltd., Accrington. Showrooms: 147 Aldersgate Street, E.C.)

Much attention has lately been centred on miniature billiard tables. Hitherto many people have considered these to be merely toys—attractive toys, but not to be regarded as giving a serious game of billiards. Now, however, professional players themselves have come to acknowledge that on properly built and truly proportioned tables the game is essentially the same as that played on full-sized tables. To those who have never played billiards on miniature tables, the correctness and the difficulty of the game come as a surprise. The angles, the pocket shots, and, in fact, all shots are just the same as on the large tables, and those interested in the game will find the miniature tables in every way worthy of serious attention. Of the several makes on the market, there are none with a higher reputation than those of Messrs. E. J. Riley, Ltd., Accrington. This firm has made a speciality of such tables, and have introduced many improvements. Their miniature tables are suitable both for private houses and for institutions, and can be obtained to place on an ordinary dining table or as a combined dining and billiard table. The latter are exceptionally convenient, as they are made to match any furniture, and while as a dining table no portion of the billiard table can be seen, they are converted in half a minute. The slate bed is very accurately finished, and stands the usual level tests excellently, while all the rubber used is of the finest black-frost proof quality, warranted not to go hard. This quality of rubber is indispensable, especially for tables in institutions, which have to stand much hard wear in rooms which are not always equably heated. The prices of the tables are very low, considering the high workmanship that characterises the firm's specialities and the equally high grade of material used throughout. Messrs. Riley issue an interesting and charmingly illustrated catalogue, which is sent post free on receipt of post-card.

### DIABETIC FOODS.

(Callard and Co., 74 Regent Street, London, W.)

We have received samples of four of the many products placed upon the market by this well-known firm for consumption by diabetics. These four specialities are Prolactic Bread, which is guaranteed to contain less than 1 per cent. of carbohydrate; Casein Lunch Biscuits, which are entirely free from starch and sugar; Gluten and Almond Biscuits, also free of the objectionable constituents; and Casoid Biscuits, of which the same holds good. We have tested the three latter and find no reaction for starch. As regards the taste of these preparations, the difficulty of rendering a diabetic diet palatable has been to a great degree met: that is, the bread and biscuits have no obnoxious taste, though, of course, they do not excite in the palate the gratifying sensations produced by similar confections made in the usual way. The number of other diabetic foods prepared by this firm, the clean and careful way in which they are presented for sale, and the reputation they have already acquired, are sufficient evidence that those who are confronted with the thankless task of humouring the whims of a diabetic may turn with confidence to the catalogue of Messrs. Callard's foodstuffs for such patients.

---

A new use of refrigeration is at present occupying the attention of the authorities of the Liverpool School of Tropical Medicine (says *Ice and Cold Storage*). This is the treatment of certain diseases common to tropical districts of Africa, by subjecting the sufferer to a cold and dry atmosphere. The idea of treating sleeping sickness, to which Europeans have proved very susceptible, by a cold-air method, was suggested to experts at the Liverpool School of Tropical Medicine some months ago, and a series of experiments to test the efficacy of the method was instituted. The co-operation of Sir Alfred Searle Haslam and of Sir Edward Durning Lawrence was sought in the matter, and was willingly given, and a suitable chamber was set apart for the experiments; and the prospects of a successful application of the cold-air treatment in cases of the kind are very promising.

